# Perceptions of prevalence, consequences, and strategies for managing contraband substance use in an inpatient concurrent disorders program: A qualitative study of patient perspectives and survey of clinician perspectives

**DOI:** 10.3389/fpsyt.2022.911552

**Published:** 2022-09-06

**Authors:** Liah Rahman, Holly Raymond, Bradley Labuguen, Hollie Gladysz, Katherine Holshausen, Jennifer Brasch, Michael Amlung, James MacKillop

**Affiliations:** ^1^Peter Boris Centre for Addictions Research, McMaster University and St. Joseph’s Healthcare Hamilton, Hamilton, ON, Canada; ^2^Concurrent Disorders Program, St. Joseph’s Healthcare Hamilton, Hamilton, ON, Canada; ^3^Homewood Research Institute, Hamilton, ON, Canada

**Keywords:** concurrent disorders, patient perspectives, contraband substance use, patient substance use, frontline staff perspectives

## Abstract

**Objective:**

Inpatient treatment programs for substance use disorders (SUDs) typically have an abstinence policy for patients, but unsanctioned substance use nonetheless takes place and can have significant negative clinical impacts. The current study sought to understand this problem from a patient perspective and to develop strategies for improved contraband substance management in an inpatient concurrent disorders sample.

**Methods:**

First, a qualitative study (*n* = 10; 60% female) was undertaken to ascertain perceived prevalence, impact, and patient-generated strategies. Second, an anonymous follow-up survey was conducted with unit staff clinicians to evaluate the suggested strategies.

**Results:**

Patients reported that contraband substance use was present and had significant negative consequences clinically. Recommendations from patients included more extensive urine drug screening, the use of drug-sniffing dogs, and direct contingencies for contraband use. Nineteen staff competed an anonymous follow-up questionnaire to evaluate the viability of these strategies, revealing variable perceptions of feasibility and effectiveness.

**Conclusion:**

These findings emphasize the adverse consequences of contraband substance use in addiction treatment programs and identify patient-preferred strategies for managing this challenge.

## Introduction

The annual prevalence of substance use disorders (SUDs) in Canada is 3.0–3.8% (1, 2) and 21.6% of Canadians experience a SUD in their lifetime (3). SUDs are commonly comorbid with other psychiatric conditions and, in contrast to the term dual-diagnosis, individuals with a SUD and another psychiatric condition are referred to as concurrent disorder patients in Canada (4). Annually, 1.2–1.7% of the Canadian population age 15 and above meet criteria for a concurrent disorder. These individuals are disproportionately represented in the mental healthcare system (1, 2, 4). Concurrent disorders are associated with several negative health outcomes and high utilization of the healthcare system, particularly emergency services and inpatient treatment programs (4–6). Concurrent disorders have also been found to be associated with more complex healthcare needs, and potentially more severe substance misuse when compared with those without a comorbid psychiatric diagnosis (7).

Treatment programs for SUDs typically adhere to an abstinence-based policy for patients while in care, but use of alcohol, tobacco cannabis, and other illicit substances nonetheless takes place (5, 6, 8, 9). This is consistent across substance use treatment settings and other health care settings, such that non-sanctioned psychoactive substance use is prohibited while on clinical grounds (10). However, precise management strategies and appropriate administrative response is at times unclear and inconsistent (10). In a 2015 study, 44% of participants who reported using illicit drugs had also used illicit drugs while in the hospital (5). Grewal et al. (6) found similar results, such that 43.9% of participants who used illicit drugs also reported using illicit drugs in the hospital in their lifetime. Contraband substance use of tobacco, alcohol, cannabis, and illicit drugs is a persistent issue in clinical settings, posing a risk not only to the patient, but also to other patients, staff, and visitors (5, 6, 8). Patients who use illicit drugs are more likely to be discharged prior to completing treatment due to non-compliance with hospital policies, resulting in negative health outcomes and greater rates of readmissions (4–6).

Research on contraband substance use in mental healthcare settings is limited, with few effective solutions established. The individual impact contraband substance use has on patients in treatment is also not well explored. The present study was initiated to address contraband substance use in an acute concurrent disorders inpatient program at St. Joseph’s Healthcare Hamilton West 5th campus (SJHH-W5) in Hamilton, Ontario with the goal of providing a safer environment for patients and staff while at the hospital. Similar to other clinical settings, contraband substance use is a challenge at SJHH-W5, but the impact of the problem is not well understood. Despite a well-established illicit substance management policy, substance use is known to take place, demonstrating a need for an effective solution. Both staff and patients have expressed negative feelings about the use of substances in the hospital and the need for more effective management strategies. To better understand the scope of the problem, the impact on patients, and develop strategies for improved contraband substance management and patient experience, we conducted a qualitative study with SJHH-W5 inpatients and then a follow-up survey with unit staff. Specifically, the study had three goals: (1) to understand patient perspectives on contraband substances; (2) to solicit patient-recommend strategies for improving management of contraband substances; (3) to evaluate the strategies identified with a follow-up staff survey.

## Study 1

### Materials and methods

#### Participants and setting

Participants were recruited from a 25-bed acute concurrent disorders inpatient unit at SJHH-W5, between July 2018 and October 2018. All patients on the unit were invited to participate. Inclusion criteria were: (1) > 18 years of age; (2) able to provide informed consent; (3) fluent in English; (4) minimum stay of 7 days. The last criterion was to both ensure psychiatric stability and that all participants had ample exposure to the environment on the unit. Eleven participants expressed interest in the study and were enrolled, but one participant was discontinued for uncooperative behavior, resulting in a sample of 10 (Table 1). All participants had privileges that allowed them to leave the inpatient unit; 70% had privileges permitting them to leave hospital property for extended periods of time with prior approval. Substance use is prohibited within SJHH-W5th and on hospital grounds, including use of tobacco, alcohol, and cannabis. If contraband substances are found on hospital grounds, the substance is confiscated and police are called to collect the substance. It is important to note that police were only contacted about the individual who brought the contraband substance to the hospital if other people were being put at risk as a result.

**TABLE 1:** Participant characteristics.

Characteristic	Mean	*SD*	%
Age (years) Range	31.9 22–58	10.8	
Sex at birth			60% female
Years of education range	13.8 12–17	1.8	
Pass level			
1			0%
2			10%
3			20%
4			70%
Alcohol use (AUDIT score)	8.4	7.6	70%
Cannabis use (CUDIT score)	11.0	9.1	80%
Illicit drug use (DUDIT score)	27.0	15.4	80%
Tobacco use (11)			80%
NIDA-modified ASSIST (any use)			
Cocaine			40%
Methamphetamine			20%
Sedatives			70%
“Street” opioids			40%
Prescription opioids			30%
Prescription stimulants			10%
Depressive symptoms (PHQ9)	9.7	4.4	
Anxiety symptoms (GAD7)	12.7	5.4	
Psychosis symptoms (PQ16)	5.3	3.3	

Key: Pass level: 1, restricted to unit; 2, restricted to hospital, cigarette breaks only; 3, day pass privileges; 4, day and weekend pass privileges; AUDIT, Alcohol Use Disorders Identification Test (12); CUDIT, Cannabis Use Disorder Identification Test (13); DUDIT, Drug Use Disorders Identification Test (14); NIDA-modified ASSIST, National Institute of Drug Abuse modified Alcohol, Smoking and Substance Involvement Screening Test; PHQ, Patient Health Questionnaire (15); GAD, Generalized Anxiety Disorder Assessment (16); PQ, Prodromal Questionnaire (symptoms associated with psychosis) (17).

#### Procedures and assessments

The research study was advertised on the unit *via* posters and announcements at inpatient group meetings by research staff (L.R.). Interested participants were contacted and assessed for eligibility. If eligible, informed consent was obtained and an interview was scheduled. Each participant completed an individual 21-question semi-structured qualitative interview with a qualified research assistant (L.R.). The interviews were up to 45-min in length (*M* = 27 min) and were audio recorded for transcription. Participant responses were iteratively reviewed by the authors to identify common themes throughout data collection. The study adopted a semi-structured framework, such that all participants were asked the same core 21-questions (available upon request), but further individualized questions and prompts were used to explore themes that emerged throughout data collection. The major goal of the interviews was to assess each participant’s experience with contraband substance use at the hospital during their stay on the unit. The interviews also assessed how those experiences had affected their stay at the hospital, impacts on their recovery, and any suggestions they had for staff to better prevent contraband substance use. For descriptive purposes, participants completed self-report assessments of demographics, mental health symptoms and substance use behavior (Table 1). The study was approved by the Hamilton Integrated Research Ethics Board (Protocol #5015).

#### Data analysis

All interviews were double transcribed verbatim to ensure accuracy. Qualitative content analysis and open coding modeled data analysis (18). The semi-structured interview items were used to guide initial development of a thematic coding scheme (19, 20). Each transcript was first read from start to finish (L.R. and C.M.) to begin identifying major themes and further develop the coding scheme. With the major themes identified, transcripts were reviewed repeatedly, and individual lines of data were coded and categorized based on the semi-structured interview items. The coding scheme continued to develop throughout data analysis, such that codes were redefined or merged, and new codes and sub-codes were created as new themes emerged. Researchers met regularly with each other, and the larger team throughout code development to discuss and define emerging themes, review the coding structure, and to negotiate discrepancies between coders. When both researchers and the larger team agreed that all major themes had been identified within the data, the coding scheme was complete. Upon completion of the final coding scheme each transcript was again reviewed line-by-line to ensure that all data had been coded according to the final scheme. Data were sorted and themes were quantified based on frequency of endorsement within and across transcripts. Upon completion of data analysis, codes were merged into three overarching themes. The themes were developed to best represent the information and opinions shared by the sample population. These methods are generally consistent with those recommended by the Cochrane Handbook (21), although this study did not solicit feedback from participants on the findings of the interviews throughout data analysis.

## Study 1 results

### Contraband substance use on hospital grounds and in the hospital

Use of tobacco cigarettes, e-cigarettes, cannabis, and other vaping devices is not permitted anywhere at SJHH-W5. Patients, staff and visitors must go beyond the perimeter of hospital property before using a cigarette, e-cigarette, or vape. Despite this policy, 90% of participants reported frequent and persistent tobacco use on hospital grounds. Individuals were frequently seen smoking tobacco cigarettes outside of the outpatient entrance of the hospital. Most participants (80%) described a high frequency of cannabis use on hospital grounds. Cannabis is used outside on hospital grounds where individuals commonly smoke tobacco cigarettes, as well as near the perimeter of property near a wooded area. The majority of participants (60%) reported that individuals were using illicit drugs on hospital grounds, but did not have many specific examples. See Table 2 for specific examples of contraband substance use on hospital grounds.

**TABLE 2:** Direct quotes about contraband substance use on hospital grounds and in the hospital.

Theme	Examples	# of Pts.
Tobacco use on hospital grounds	***P008:** “I do smoke on hospital grounds*…” ***P011:** ”And then obviously on the grounds at like the outpatient entrance and main entrance”* ***P004:** ”People were smoking out in the yard, ‘cause there’s a blind spot in the camera*… *there’s a cigarette up there, there’s a lighter that’s hidden in the dirt.”*	9/10
Marijuana use on hospital grounds	***P004:** “Oh yeah, I’ve used marijuana too.”* ***P011:** ”Oh, okay, so I’ve smoked weed on–I don’t know if it’s the grounds though*…*but it’s like the foresty area.”*	8/10
Illicit drug use on hospital grounds	***P003:** “I haven’t seen it, but I’ve seen the result*… *heroin I’m pretty sure he was doing.”* ***P008:** “.I can tell that they’re using and I know how they’re using, and what they’re using.”* ***P008:** “I haven’t seen it, but I know it’s happening, and it’s a very specific person too.”* ***P008:** “.the person is using methamphetamines still and talking about it. He eats it*…” ***P008:** ”There’s actually like a group that strays off from the smoking area in the corner of the parking lot that’s pretty heavy into anything and everything.”*	6/10
Tobacco use in the hospital	***P001:** ”No, but I’ve heard people say that they do, and I was even given advice [laughs] that if you turn the shower on and go near the drain it helps get rid of the smell”* ***P003:** “.I don’t know who’s smoking in the hallway, but it’s still happening. There’s ashes in there, and carrying in on my shoes and making me dirty. The smell makes me feel sick.”* ***P004:** “I was smoking in my room”* ***P008:** “It’s more on the stairways and on the locked units upstairs—they smoke.”* ***P010:** ”Like, I won’t lie I did it in my room. Like a lot, especially in the morning.”* ***P010:** ”So there were times when I had to smoke in my bathroom, because you know what? They wouldn’t let me out until 8:30 and I could not go and sit in the breakfast room dealing with all those people, you know, before I had my smoke.”* ***P011:** “I know that people smoked cigarettes in their room on the ward.”* ***P011:** ”I’ve smelled it, and I’ve seen like cigarette butts, and people walking out of stairwells particularly in the gym. The gym is like a big one, not like the fitness room, but like the basketball court kinda place.”*	6/10
Marijuana use in the hospital	***P003:** “I haven’t seen them, but one day I smelled some marijuana on my way to a meeting actually, an N.A. meeting, and it was a trigger for me*…” ***P004:** “.they caught me with five joints*…” ***P006:** ”No, but I’ve seen it in the unit.”* ***P010:** “I heard about somebody vaping. So, yeah, I guess they did use marijuana on the unit.”* ***P011:** “No, but I’ve smelled it.”*	6/10
Illicit drug use in the hospital	***P003:** “So, one other person, I believe that he is a heroin user, and I saw him in the hallway upstairs on the way to a meeting and his eyeballs rolled up in his head*…*and he was about to fall over, and he went into arrest”* ***P004:** “In the hospital I seen [sic] a guy use heroin and got narked*…” ***P004:** it was this new stuff, it’s called—it’s called purple fentanyl. It’s actually purple, they put–they must put food coloring in it or something’, but it’s more lethal than anything. It’s called purple popcorn they call it. It’s a mix of heroin and fentanyl.”* ***P008:** ”I tried to play a game of chess against the man, and he had to leave to go use*…” ***P009:** “I know it’s happening, but I don’t see it.”* ***P010:** “I didn’t see anybody, I heard and I seen [sic] the after effects*…” ***P011:*** “…*there are other people that I know have used drugs and smoked cigarettes on the ward, like in their rooms and they haven’t gotten discharged.”* ***P011:** “I saw someone with a meth pipe putting into their bag in the ward when they were like–they kind of sneaked [sic] it into their bag. When their bag was getting searched, like they snuck it into in between a book*…” ***P011:** “I had been told that they were like snorting their Wellbutrin, like their prescription, but I’d never seen it.”*	6/10
Intoxicated patients in the hospital	***P002:** “I think they go out on their passes and use substances. And come back to the unit all high or whatever. I just witnessed an overdose this morning, so there must be drugs on the unit right now.”* ***P003:** ”And the other thing I noticed is someone is abusing their night time medication to get high, and then putting off going to sleep and almost falling over and banging his head off the floor.”* ***P009:** “I can clearly see that they’re on something.”* ***P010:** “I seen [sic] somebody who was drunk after they came back from a pass*…”	6/10

The # of PTs is the number of participants who endorsed each theme.

Within the hospital, the majority of participants (80%) reported that patients smoke cigarettes. Participants reported that patients smoke cigarettes in their rooms, in bathrooms, or in other common areas, such as the patient gym and stairwells. Some participants admitted to smoking cigarettes in their rooms. They had received advice from other patients on how to use cigarettes on the unit without being noticed. Most participants (70%) described extensive exposure to cannabis use in the hospital. Participants reported smelling cannabis in hallways and stairwells, suggesting that it had been used indoors. Many participants reported being offered cannabis and seeing other patients with cannabis products while on the unit. Most participants (70%) reported that illicit drug use occurs within the hospital. Participants reported seeing or hearing about heroin, fentanyl, and methamphetamine use in the hospital. Materials associated with illicit drug use have been found by participants on the unit. The majority of participants (60%) described encounters with intoxicated patients within the hospital. It was reported that patients used contraband substances within the hospital or returned from an off-campus pass visibly intoxicated by alcohol, cannabis, or illicit drugs. Two participants described a distressing incident of witnessing a co-patient experience an opioid overdose while in the hospital. See Table 2 for specific examples of contraband substance use in the hospital.

### Impact of contraband substance use on patients

One of the main concerns when addressing this topic is the impact on patient wellbeing. The majority of participants (70%) reported being negatively impacted by the presence of contraband substances at the hospital. Seeing other patients intoxicated, being offered contraband substances, and knowing that patients were using at the hospital was described as “triggering” and frustrating by participants. Participants reported strong cravings, anger toward others, and engaging in substance seeking behavior that interfered with their own recovery. Participants described feeling unsafe in the hospital knowing that contraband substances were present. See Table 3 for specific examples of how participants were affected by contraband substance use in the hospital.

**TABLE 3:** Direct quotes about how participants responded to contraband substances at the hospital.

Theme	Examples	# of Pts.
Triggering events	***P001:** ”.just that I have a hard time knowing that other people are, when I would definitely like some [alcohol]”* ***P001:** ”Just triggers, because I want it. I’m still struggling with suicide thoughts since I’ve been here, and that was always my coping mechanism, was to use cocaine. So, it just makes me want it even more, or I get depressed, because I feel like that and it just makes all the feelings come back to me”* ***P002:** ”It’s discouraging because like I’ve tried my hardest to be clean and sober*…*and it’s just really triggering when you know someone comes in off the unit drunk.”* ***P003:** ”It put me in a bad mood. It made me think about what I’m gonna do when I get out of here and how I’m gonna avoid that.”* ***P008:** ”Terribly, because the person using methamphetamines still and talking about using it reminder me of when I was first here.”* ***P008:** ”But if someone’s offering shatter, which someone did–that fucked me right up, ‘cause it’s like, I’m literally here because of suicide and shatter.”* ***P009:** ”It was triggering. I was offered alcohol, so that was hard for me, ‘cause I–I was close to drinking. So, yeah. Being offered–not just seeing it wasn’t bad, but being offered it was bad for me.”* ***P010:** ”It was triggered–it triggered me. It made me very upset. It made me, you know, feel like they were wasting the hospital sta–like the hospital’s time. It made me feel like they didn’t really want to be clean and recovered, like they would just outright say, “oh, when I get out of here, you know, I’m going right back to doing what I was doing before.”* ***P010:** ”Yeah, well it affected me personally, it was like, when I was having like a bad day, and I was fighting my urges or my cravings, and then hearing somebody talk about it*…*like I was like, ‘no, I don’t–I don’t want to go back to that.’*…. *So it was hard.”*	7/10

The # of PTs is the number of participants who endorsed each theme.

### Prospective strategies to prevent contraband substance use

Specific examples of strategies suggested by participants are presented in Table 4. It is routine on the unit for frontline staff to search belongings brought in by patients or visitors. However, several participants (70%) described how patients have been able to smuggle contraband substances such as alcohol, marijuana, and illicit drugs onto the unit by hiding them in their clothing, purses, and backpacks. Many participants suggested that increasing the thoroughness and frequency of clothing and belonging searches on patients would be an effective way to reduce contraband substances from entering the hospital.

**TABLE 4:** Direct quotes regarding suggested strategies for staff to prevent contraband substance use in the hospital.

Theme	Examples	# of Pts.
Drug sniffing dogs	***P001:** “I think that would make me feel safe actually”* ***P001:** “I really have no intention, nor have I thought about bringing anything to the unit, but I would certainly be a hell of a lot more scared, and I think it would scare other people.”* ***P003:** ‘That’s a good idea.”* ***P004:** “If you had drug sniffing dogs, people would—then people would smarten the fuck up. That’s—that is honestly a huge action.”* ***P008***: *“Maybe get a drug dog—hire a single person to bring a drug dog once a month.”* ***P010***: *“I guess it would depend on how rampant the drugs were*…*if they could honestly not find the drugs and they knew for a fact that they were there.”* ***P011***: *“No I would not feel good about that*…*I think that that creates a fear and it creates an environment in which people do not want to be honest and open about their use.”*	8/10
Body/Clothing searches	***P001:** “They could maybe do searches on people, kinda like they do at a concert, like obviously they can only touch so many areas, but maybe check pockets and take off your shoes.”* ***P004***: *“Body searching”* ***P006***: *“If they have a change of clothes*… *they could change into a new pair of clothes so they could have their other one checked.”*	7/10
Increased searching of belongings	***P002***: *“Again just checking bags that are being brought in*…” ***P003:*** “…*they need to look for drugs in their rooms or on their personal, and in places where you might not look for stuff, ‘cause these people know how to hide stud, say behind here [points to electrical outlet].”* ***P008:*** “…*if you enter in one of the units, like be admitted, I think they should make you empty out your pockets, take off your shoes, take our your socks–you know, everything but your underwear, and then as humiliating as that it, it would stop a lot of flow of drugs.”* ***P009:** “Every time we go out on a pass I think we should be searched.”* ***P010***: *“More thorough checks. I know in forensics they’re required to empty their pockets.”*	7/10
Mandatory drug tests and breathalyzers	***P002:*** “…*I know we have a breathalyzer here on the unit, so they can use that.”* ***P008:** “Make ‘em pee in a cup. Even if they go for a smoke and they come back smelling of weed, make ‘em pee in a cup.”* ***P009***: *“And maybe mandatory drug tests for this unit*…” ***P010***: *“Like, so I really feel like nurses should have the initial test strips and do the initial one right in front of you to show that you did or didn’t use, and then take the appropriate steps. So the test strips, like drug screening tests. And I believe they’re fairly cheap, like every methadone clinic does it that way.”*	6/10
Consequences for repeat offenses	***P003***: *“You know, it’s sort of like you don’t give them three chances [laughs], you know, once they–they do it, then they need to be restricted, basically so that they can’t do it.”* ***P011***: *“I think they can be more disciplinary with repeat offenders”* ***P008***: *“*…*just take away their smoking privileges.”* ***P011:** “Like if someone specifically is known to use drugs, alcohol, or smoke tobacco, like on the unit or within the hospital, I think that more measures could be taken with those specific people*…” ***P011***: *“I think people should be discharged if they’re unwilling to get well.”*	5/10
Open dialogue	***P002:** “So, just like being open and transparent and honest from the nurses and from the patients would be most helpful.”* ***P004***: *“.discussion with nurse.”* ***P008***: *“Provide more awareness of punishments.”* ***P008***: *“So, you know, if you tell everybody, like, ‘hey, this is the new protocol”, at one of the group meetings or whatever*…” ***P011***: *“*…*If we maybe had like a group for like every time someone–like if we have a new group of people or something, if they have to attend something that’s kind of like a–almost like a harm reduction for the unit. Maybe where they’re told like, “hey, here are the reason why we don’t want this. It’s not just because we–we wanna be mean.so that other people are maybe more concerned about the wellbeing of other patients.”* ***P011***: *“As well as maybe telling them–urging them to be honest with doctors and staff about their use, as opposed to hiding it, and kind of creating an open door policy about it, because ultimately I don’t think being too harsh too soon works.”* ***P011***: *“I think if you come to staff, or your doctors and stuff and you’re being honest about your use, they should come up with like a plan specific to you and your use and I think they should also urge people that if they are going to use drugs or alcohol to keep it off the ward and to keep it somewhere else maybe.”*	5/10

The # of PTs is the number of participants who endorsed each theme.

The majority of participants (70%) reported that utilizing drug sniffing dogs on the unit would be effective in not only locating contraband substances, but also preventing them from being brought in. Participants reported that drug sniffing dogs would make them “*feel safe*” (P001) and would be “*a good idea*” (P003) for the hospital to implement. One participant noted in reference to drug sniffing dogs that “*I really have no intention*…*about bringing anything to the unit, but I would be a hell of a lot more scared, and I think it would scare other people.*” (P001), suggesting a deterrent effect. Two participants reported that drug sniffing dogs may erode trust between patients and staff.

Urine drug screens (UDS) and exhaled breath alcohol tests (i.e., breathalyzers) are currently administered on an as needed basis if a patient is suspected of being intoxicated. Several participants (60%) recommended a more routine system where patients are required to complete a UDS or breathalyzer on a regular basis or whenever they return to the unit after a pass.

Participants expressed an understanding that other patients make mistakes and will use or traffic contraband substances while in treatment. However, 50% of participants suggested that there should be an escalating system of consequences for a patient who persistently traffics or uses contraband substances at SJHH-W5. Participants felt that if a patient repeatedly traffics substances, they should lose their privileges or be discharged for the wellbeing of other patients.

## Study 2

### Materials and methods

One of the main goals of this study was to evaluate the strategies suggested by participants of Study 1. A follow-up staff assessment was initiated to evaluate some of the strategies that had been suggested by patients for hospital staff to better prevent contraband substance use. Clinical staff on the unit were invited to complete an anonymous survey to evaluate the feasibility and potential efficacy of 10 of the suggested strategies.

### Participants

All clinical staff on the unit (i.e., full-time and part-time nurses and addiction counselors) were invited to volunteer to complete the survey anonymously. Nineteen participants completed the survey.

### Procedures

Based on study 1, 10 strategies suggested by participants were selected for staff evaluation: (1) body pat-downs on patients when patients re-enter the unit; (2) search patient’s pockets and shoes when they re-enter the unit; (3) search personal belongings when a patient re-enters the unit; (4) drug sniffing dogs on the unit; (5) weekly random drug tests and breathalyzers on patients; (6) search belongings of patient’s visitors; (7) regular searches of patient lockers; (8) move patient lockers onto the unit (“Mailbox” style to allow staff to see the inside of lockers from one side at all times); (9) escalating consequences for repeated offenses; and (10) mandatory groups to discuss rules and consequences of bringing contraband substances onto the unit. The strategies were chosen based on the patient data and clinical priorities. The study was announced at the weekly staff huddle, and paper surveys were made available in the staff meeting room on the unit for 1 month. Staff were invited to complete the survey anonymously by rating each strategy on a 5-point Likert-type scale for feasibility (very feasible, somewhat feasible, unsure, somewhat infeasible, very infeasible) and efficacy (very effective, somewhat effective, unsure, somewhat ineffective, very ineffective). To maintain anonymity, a secure box was placed on the unit to collect the surveys. If staff wanted to participate, they were invited to complete the paper survey and place it in the secure box before the end of the 1 month period. The surveys were gathered from the box by research staff at the end of each workday. All procedures were approved under the same REB protocol.

## Study 2 results

**FIGURE 1:**
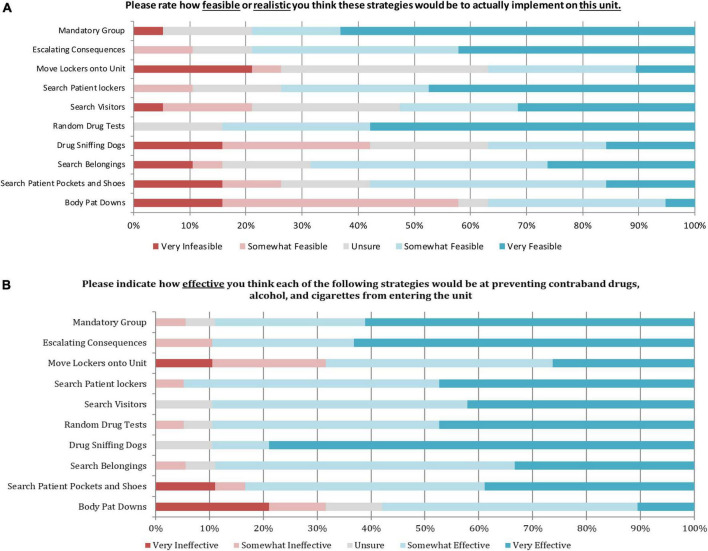
Results from the frontline staff survey on the perceived feasibility of the 10 suggested strategies (*N* = 19). **(A)** Depicts feasibility. **(B)** Depicts effectiveness.

The results from the survey are presented in Figure 1. Conducting weekly random drug tests and breathalyzers was rated as the most feasible option, with 57.9% of participants rating it as “very feasible” and 26.3% as “somewhat feasible.” Some participants were unsure of the feasibility (26.3%), however, 0% felt that this would be infeasible on the unit. Completing body pat-downs on patients when they re-enter the unit was rated as the least feasible option, with 42.1% of participants rating it as “somewhat infeasible” and 15.8% rating it as very infeasible. Despite that, some staff did feel that body pat-downs were feasible with 31.6% rating the strategy as “somewhat feasible” and 5.3% rating it as “very feasible.” One individual was unsure of the feasibility (5.3%).

Drug sniffing dogs were rated as the most effective strategy with 78.9% of participants rating it as “very effective” and 10.5% reporting it as “somewhat effective.” While some participants were unsure of the effectiveness (10.5%), 0% felt that this would be an ineffective strategy for preventing contraband substance use at SJHH-W5. Patients currently have access to a private locker outside of the unit. Moving patient lockers onto the unit was rated as the least effective strategy with 21.1% of participants rating it as “somewhat ineffective” and 10.1% as “very ineffective.” However, many participants did feel that this would be an effective strategy, as 42.1% rated the strategy as “somewhat effective” and 26.3% felt that it would be “very effective.” Completing body pat-downs on patients when they re-enter the unit was also seen as one of the least effective strategies, as 21.1% of participants believed it would be “very ineffective,” and 10.5% felt that it would be “somewhat ineffective.” Several participants reported that body pat-downs would be effective, as 47.4% rated as “somewhat effective” and 10.5% rated as “very effective,” while others (10.5%) were unsure of the efficacy.

## Discussion

The present studies were initiated to gain patient perspectives on contraband substance use on a concurrent disorders inpatient unit, and subsequently solicit strategies from hospital staff in response to patient-generated recommendations. Despite efforts to prevent contraband substance use at the hospital, our findings from study 1 show that use of contraband substances is a significant issue that negatively impacts a patient’s stay and wellbeing. Encounters with contraband substance use at the hospital were reported by the majority of study 1 participants and were described as triggering and upsetting, and many participants reported a negative impact on their recovery. Contraband substance use frequently occurs in common areas of the hospital, including stairwells and around the outside of the building, as well as on the unit in patient rooms and washrooms. Our findings are consistent with previous survey and interview-based studies that found high rates of contraband substance use in hospitals among patients who reported using alcohol or other substances in their lifetime (5, 6, 9, 10). One study from Vancouver, Canada similarly reported that alcohol and other substance use most commonly occurs in washrooms, smoking areas, and in hospital rooms while patients were receiving acute care (6). Another study set in Ontario, Canada reported occurrences of substance use in hospital settings among individuals seeking treatment for non-substance use care. Participants reported leaving the hospital to access drugs, using substances in washrooms or post-surgical rooms, and having substance brought to them by hospital visitors (10). Though neither of the aforementioned studies focused specifically on SUD or concurrent disorder treatment settings, their findings do suggest that contraband substance use is a recurring issue across healthcare settings. Additionally, Strike et al. (10) cited a lack of clear and appropriate administrative response to the presence of contraband substances in the hospital and highlighted the need for a more patient-centered response to the issue. The present study’s focus on patient impact and perspectives hopes to close this gap.

Consistent with previous research, tobacco was commonly used both in the hospital and on hospital grounds despite tobacco-free policies (22). SJHH-W5 adheres to the Smoke Free Ontario Act, such that smoking of any kind is not prohibited within the hospital or on hospital grounds (23). However, participants reported repeated experiences with tobacco use on hospital property, in stairwells, patient rooms, and washrooms. Some participants suggested that the distance required to leave hospital property where smoking is prohibited, combined with the restrictive curfew on the unit motivates patients to smoke within the hospital. Several participants suggested that creating a smoking area on hospital grounds would eliminate the need to smoke in restricted areas, and significantly mitigate the issue. This strategy cannot be implemented, as Ontario laws do not permit smoking of any kind within public establishments, like hospitals, or on hospital property (23). Existing research suggests accessibility to smoking cessation or nicotine replacement products is a possible solution to tobacco use in the hospital, but may not be enough to significantly reduce a patient’s likelihood to smoke (24). Patients on the unit are provided with nicotine replacement products as needed, but still resort to smoking in their rooms and elsewhere in the hospital, suggesting the need for additional approaches to reduce illicit tobacco use.

In addition to tobacco use, alcohol, cannabis, and other illicit drugs were commonly used among patients at the hospital. While other studies have found similar results, few have formulated solutions to the problem. A major goal of this study was to address this gap in the research by soliciting suggestions from patients for staff to better manage contraband substance use at the hospital. Findings from study 1 demonstrated that illicit substance use is a significant issue affecting patients within the hospital. Study 1 also supported the need for further interventions and strategies to manage contraband substance use. To address this, study 2 was initiated to solicit input from frontline staff on potential management strategies suggested by patients. Participants from Study 1 provided several potential strategies based on their perspectives as patients. Our findings reflect that several participants are aware that patients can smuggle contraband substances in and out of the unit on their person or in their personal belongings. Participants reported that patients may commonly return from a pass intoxicated by alcohol or other drugs. To mitigate these behaviors, participants suggested strict policies for searching patient clothing and belongings anytime they return to the unit, as well as frequent searches of patient rooms and lockers. Frontline staff did not see this as a feasible or effective strategy, possibly due to the amount of time and resources required, and the potentially invasive nature.

Breathalyzer tests and UDSs were suggested by patients to occur on a frequent and random basis. Frontline staff also endorsed more frequent breathalyzer tests and UDSs as a feasible and potentially effective strategy, as demonstrated by the results of study 2. It is important to note that breathalyzers and UDSs are currently part of the SJHH-W5 illicit substance management policy. However, participants of study 1 reported that they happen infrequently and only when intoxication is suspected. Implementing a policy where breathalyzers or UDSs can be administered frequently and at random may deter patients from using contraband substances while staying at the hospital. Other units at SJHH-W5 are reported to implement a “marble” strategy, whereby patients are selected at random to receive a UDS and breathalyzer if their marble is selected from a bin. Implementing a similar strategy on other inpatient units may be affective in better integrating regular and random UDSs and breathalyzers as a management strategy.

One of the most popular strategies endorsed by participants was the implementation of drug sniffing dogs. This strategy was seen as a way to not only to find substances that have already been brought onto hospital grounds, but also to deter patients from trafficking contraband substances in the future. Participant’s felt that even the knowledge that drug sniffing dogs could be brought into the facility would reduce the presence of contraband substances in the hospital and increase feelings of safety among patients. Frontline staff expressed agreement with this suggestion, as most participants of study 2 believed it would be an effective strategy. Drug sniffing dogs can be utilized to efficiently and effectively identify and locate illicit substances in public settings (8, 25). Drug sniffing dogs were approved as a strategy at SJHH-W5th in September 2020 following findings from the present study. Drug sniffing dogs were brought into the hospital on two separate occasions, and a thorough search of inpatient units, public spaces, and the hospital perimeter was conducted. Following implementation, feedback was gathered *via* survey from both staff and patients present during the drug dog visits. Overall, use of drug sniffing dogs was perceived positively by both staff and patients, as both felt it to be an effective and valuable strategy that would benefit the safety of the hospital environment. The majority of staff and patient respondents agreed that the strategy was not an invasion of patient privacy and would be in support of this as on ongoing strategy.

Several considerations bear noting. Our findings were somewhat limited by a relatively small sample size. In addition, the unit is an acute treatment care setting for concurrent disorders patients with a degree of acuity and clinical complexity and, over the course of recruitment, a sizable proportion of patients were unwell or uninterested in participating. Finally, although we anticipate substantial similarities, this study had only one treatment site. All of these factors will potentially affect the generalizability to other clinical settings.

## Conclusion

This study provides rich patient perspectives on the issue of contraband substance use on an acute concurrent disorders unit. Several strategies were put forward by the patients who participated and were supported by the frontline staff who would be primarily responsible for implementing new policies. Moving forward, these strategies warrant consideration in similar care settings to create more effective contraband substance management protocols. Maintaining a safe and substance-free environment in acute concurrent disorder inpatient units, and SUD treatment settings more generally, is critical to fostering the best possible clinical outcomes and quality of care.

## Data availability statement

The raw data supporting the conclusions of this article will be made available by the authors, without undue reservation.

## Ethics statement

The studies involving human participants were reviewed and approved by Hamilton Integrated Research Ethics Board. The patients/participants provided their written informed consent to participate in this study.

## Author contributions

LR contributed to protocol development, conducted all data collection, transcription, qualitative coding, data analysis for Study 1 and 2, and wrote the manuscript. HR contributed to study conceptualization, protocol development, and manuscript revisions. BL provided clinical oversight, contributed to protocol development, and manuscript revisions. HG, KH, JB, and MA provided guidance during data analysis and contributed to manuscript revision. JM developed study concept, contributed to protocol development, provided oversight during data collection and analysis, and manuscript revisions. All authors contributed to the article and approved the submitted version.

## Abbreviations

SJHH-W5: St. Joseph’s Healthcare Hamilton West 5th Campus.
